# Splicing Machinery Facilitates Post-Transcriptional Regulation by FBFs and Other RNA-Binding Proteins in *Caenorhabditis elegans* Germline

**DOI:** 10.1534/g3.115.019315

**Published:** 2015-08-11

**Authors:** Preston Novak, Xiaobo Wang, Mary Ellenbecker, Sara Feilzer, Ekaterina Voronina

**Affiliations:** Division of Biological Sciences, University of Montana, Missoula, Montana 59812

**Keywords:** germline, splicing factor, RNA-binding protein, stem cells

## Abstract

Genetic interaction screens are an important approach for understanding complex regulatory networks governing development. We used a genetic interaction screen to identify cofactors of FBF-1 and FBF-2, RNA-binding proteins that regulate germline stem cell proliferation in *Caenorhabditis elegans*. We found that components of splicing machinery contribute to FBF activity as splicing factor knockdowns enhance sterility of *fbf-1* and *fbf-2* single mutants. This sterility phenocopied multiple aspects of loss of *fbf* function, suggesting that splicing factors contribute to stem cell maintenance. However, previous reports indicate that splicing factors instead promote the opposite cell fate, namely, differentiation. We explain this discrepancy by proposing that splicing factors facilitate overall RNA regulation in the germline. Indeed, we find that loss of splicing factors produces synthetic phenotypes with a mutation in another RNA regulator, FOG-1, but not with a mutation in a gene unrelated to posttranscriptional regulation (*dhc-1*). We conclude that inefficient pre-mRNA splicing may interfere with multiple posttranscriptional regulatory events, which has to be considered when interpreting results of genetic interaction screens.

Whole-genome synthetic interaction screens are used widely to identify functional partners of genes of interest. Large-scale analyses performed in *Caenorhabditis elegans* suggest that the majority of genes fail to produce a phenotype when singly depleted (Kamath *et al.* 2003), partially because of genetic redundancy. Synthetic phenotypes produced by simultaneous depletion of two genes and not observed in either single mutant often are interpreted as an indication of functional connections between genes. Synthetic interaction screens are a valuable tool to probe the complex regulatory networks. Here, we use synthetic interaction screen to identify factors contributing to regulation of the network that maintains the balance between stem cell proliferation and differentiation in the germline.

*Caenorhabditis elegans* germ cells undergo a stereotypical developmental program that ends in the production of mature gametes prepared for fertilization (Pazdernik and Schedl 2013). The germline functions as an assembly line, where stem cell proliferation and self-renewal occurs at the distal region in the stem cell niche supported by the activation of GLP-1/Notch signaling pathway (Kimble and Crittenden 2007). Meiotic differentiation is triggered as the germ cells are displaced from the niche (reviewed in Kershner *et al.* 2013). As germ cells move proximally, they transit through the stages of meiotic prophase and ultimately form fully differentiated gametes (sperm or oocytes). In a *C. elegans* hermaphrodite, germ cells of late larva develop along the male pathway and form sperm, and germ cells of the adult develop along the female pathway, forming oocytes. The balance between stem cell self-renewal and differentiation must be carefully maintained to support tissue development and maintenance. Regulation of stem cell proliferation and differentiation is characterized by multiple redundancies, feedback and feed-forward modules, and is also tightly integrated with regulation of germline sex determination.

In *C. elegans* germline, posttranscriptional mechanisms play a major role in the regulatory network determining the extent of germline proliferation (Hansen and Schedl 2013). For example, the PUF domain RNA-binding proteins FBF-1 and FBF-2 (collectively referred to as FBFs) maintain germline stem cell fate and prevent meiotic differentiation (Zhang *et al.* 1997; Crittenden *et al.* 2002; Lamont *et al.* 2004). FBFs repress differentiation-associated mRNAs, which include genes promoting differentiation/meiotic entry, genes supporting meiotic processes, and genes associated with spermatogenesis (Crittenden *et al.* 2002; Thompson *et al.* 2005; Merritt and Seydoux 2010). In addition to the FBFs, several splicing factors contribute to the regulation of the balance of proliferation and differentiation (Belfiore *et al.* 2004; Mantina *et al.* 2009; Kasturi *et al.* 2010; Kerins *et al.* 2010; Zanetti *et al.* 2011; Wang *et al.* 2012). The data to date suggest that an overall decrease in spliceosomal activity may induce overproliferation of germline, although the mechanism of splicing factor regulatory contribution remains unknown.

Germ cell differentiation into sperm or oocytes depends on the germline sex determination pathway. The developmental switch of *C. elegans* germline from spermatogenesis to oogenesis also is under posttranscriptional regulation that determines the number of sperm produced before the hermaphrodite switches to oogenesis (Francis *et al.* 1995; Crittenden *et al.* 2002; Zanetti and Puoti 2013). This decision depends on the relative abundance of proteins promoting male fate (such as FOG-1, FOG-3, and FEM-3) and the proteins promoting female fate (such as TRA-2 and TRA-3) (reviewed in Zanetti and Puoti 2013). In the L3/L4 larval stages, when *C. elegans* hermaphrodites produce sperm, proteins promoting male fate, including FOG-1, are expressed, whereas the female fate-associated *tra-2* is translationally repressed. In the adult hermaphrodite, germ cells switch from spermatogenesis to oogenesis in response to the translation of the female fate mRNA *tra-2* and translational repression of the male fate mRNA *fem-3* (Ahringer and Kimble 1991). FOG-1 is one of the germline regulatory proteins necessary for sperm development and is an RNA-binding protein of the cytoplasmic polyadenylation element binding protein (CPEB) family (Jin *et al.* 2001b, Thompson *et al.* 2005). FOG-1 promotes proliferation and spermatogenesis during male as well as hermaphrodite larval development (Barton and Kimble 1990, Thompson *et al.* 2005). *fog-1* is one of the terminal regulators in the germline sex determination cascade, and loss-of-function mutations in *fog-1* cause germline feminization, which is epistatic to a number of masculinizing mutations (reviewed in Zanetti and Puoti 2013).

Several factors coordinately regulate both the germline stem cell proliferation/differentiation switch and the spermatogenesis/oogenesis transition. For example, in addition to promoting stem cell renewal, the FBF proteins also repress protein production from *fem-3* and *fog-1* mRNAs (Zhang *et al.* 1997, Thompson *et al.* 2005). Indeed, *fbf-1fbf-2* double mutant animals fail to make oocytes, which results in germline masculinization (Crittenden *et al.* 2002). *fog-1* mRNA is a direct target of FBFs, its 3-prime untranslated region (3′UTR) contains FBF binding sites that are necessary for silencing FOG-1 protein expression in the mitotic germ cells (Thompson *et al.* 2005). Similarly, loss–of-function mutations in a number of splicing factors cause masculinization of the germline, possibly through regulation of *fem-3* translation (Graham and Kimble 1993; Puoti and Kimble 1999, 2000; Belfiore *et al.* 2004; Kawano *et al.* 2004; Konishi *et al.* 2008; Mantina *et al.* 2009; Kasturi *et al.* 2010; Kerins *et al.* 2010; Zanetti *et al.* 2011; Wang *et al.* 2012).

Splicing of pre-mRNA proceeds through the activity of the spliceosome, which is a large and dynamic protein−RNA complex that assembles on the mRNA in a characteristic step-wise fashion while progressing from recognition of 5′ and 3′ intron boundaries to eventual intron excision (Lee and Rio 2015). Efficient splicing is critical to generate a translatable open reading frame, and additionally plays a role in regulating multiple aspects of RNA metabolism including nuclear export, mRNA stability, localization, and translational activity (Nott *et al.* 2003; Hachet and Ephrussi 2004; Popp and Maquat 2014).

In this study, we set out to identify cofactors of FBF-2 by using genetic interaction screening. FBF-1 and FBF-2 are redundant, and although inactivation of a single gene does not produce a phenotype, simultaneous inactivation of both *fbfs* leads to a loss of germline stem cells and sterility. Previously, we reported that FBF-1 and FBF-2 repress their target mRNAs using distinct mechanisms (Voronina *et al.* 2012), which now allows to identify genes required for FBF-2 function. Knockdown of such genes results in sterility only when *fbf-1* function is compromised but not when *fbf-2* function is compromised. In this study, we find that knockdown of splicing factors disrupted FBF function as well as compromised the function of at least one other RNA-binding protein. We conclude that in addition to their established role in mRNA biogenesis, the splicing factors act more broadly to maintain efficient translational control of germline mRNAs.

## Materials and Methods

### Nematode culture

*C. elegans* strains (Table 1) were derived from Bristol N2 and cultured according to standard protocols (Brenner 1974) at 15°, 20°, or 24° as indicated.

**Table 1:** Nematode strains used in the study

Genotype	Transgene Description	Strain	Reference
Transgenes: GFP::H2B::3′UTR			
* rrf-1(pk1417) axIs1772 [pCM1.90] I*	*pie-1* prom::GFP::H2B::*fog-1* 3′UTR	UMT193	This study
* rrf-1(pk1417) axIs1772 [pCM1.90] I*; *fbf-1(ok91) II*	*pie-1* prom::GFP::H2B::*fog-1* 3′UTR	UMT191	This study
* rrf-1(pk1417) axIs1772 [pCM1.90] I*; *fbf-2(q738) II*	*pie-1* prom::GFP::H2B::*fog-1* 3′UTR	UMT194	This study
Mutant strains; no transgene			
* dhc-1(or195) I*	−	EU828	Hamill *et al.* 2002
* rrf-1(pk1417) I*	−	MAH23	Kumsta and Hansen, 2012
* rrf-1(pk1417) I*; *fbf-1(ok91) II*	−	UMT186	This study
* rrf-1(pk1417) I*; *fbf-2(q738) II*	−	UMT203	This study
* fog-1(q523) rrf-1(pk1417) I*	−	UMT220	This study

### RNA interference (RNAi)

RNAi was performed by feeding method, RNAi constructs were derived from Source BioScience RNAi library (Kamath and Ahringer 2003); all clones were verified by sequencing. Empty vector pL4440 was used as a negative control throughout the experiments. Three colonies of freshly transformed RNAi plasmids were combined for growth in LB/Carbenicillin media for 4 hr and induced with 10 mM Isopropyl β-D-1-thiogalactopyranoside for 2 hr more at 37°. RNAi plates (NNGM plates containing 75 μg/mL carbenicillin and 0.4 mM Isopropyl β-D-1-thiogalactopyranoside) were seeded with the pelleted cells. RNAi treatments for genetic interactions with *fbf-1*, *fbf-2*, and *fog-1* were performed by feeding the L1 hermaphrodites synchronized by bleaching with bacteria expressing double-stranded RNA for 70 hr at 24° (*fbf-1*, *fbf-2*) or for 144 hr at 15° (*fog-1*). RNAi on strains expressing green fluorescent protein (GFP)-tagged histone H2B was performed at 24°.

### Assessment of sterility, masculinization, and reporter deregulation

Sterility of the treated worms was scored when no embryos were observed in the uterus at day 1 post L4. Masculinization of germlines was assessed after the treated worms were fixed, and chromatin was stained with 4′,6-diamidino-2-phenylindole (DAPI); germlines with sperm and no oocytes were scored as masculinized. Regulation of GFP::H2B::*fog-1* 3′UTR reporter was assessed by obtaining images of all germlines with identical exposure settings (2.8 sec). Epifluorescent images were acquired with an AxioCam MRm camera attached to a Zeiss Axioscop with a 63x Plan-Apochromat NA 1.4 objective using Zen Blue software (Zeiss). When expression of the fluorescent reporter was detected in the distal mitotic region, the germline was scored as “derepressing in stem cells.” To assess reporter overexpression, accumulation of nuclear GFP reporter was quantified in five transition zone nuclei per each germline and corrected to background using Zen Blue. Brightness values were normalized to the average intensity of the reporter in the *rrf-1* background following control RNAi. Image processing was performed in Adobe Photoshop CS4.

### Embryonic lethality assessment

RNAi treatments were performed at 15°. Wild-type (N2) or *dhc-1(or195ts)* animals at the fourth larval stage were placed on RNAi feeding plates and left overnight. The next day, the adult worms were transferred into a fresh RNAi plate and incubated for 5 hr before being removed from the plate. After removal of the adult worms, plates were incubated for 48 hr at 15°, and the number of unhatched eggs and larval worms on the plate was scored. Embryos were scored as dead or arrested if they didn’t hatch after at least 2 d after being deposited on the plate.

### Data availability

Strains are available upon request.

## Results

### Splicing factor RNAi results in enhanced synthetic sterility with mutants of either *fbf-1* or *fbf-2*

**Figure 1:**
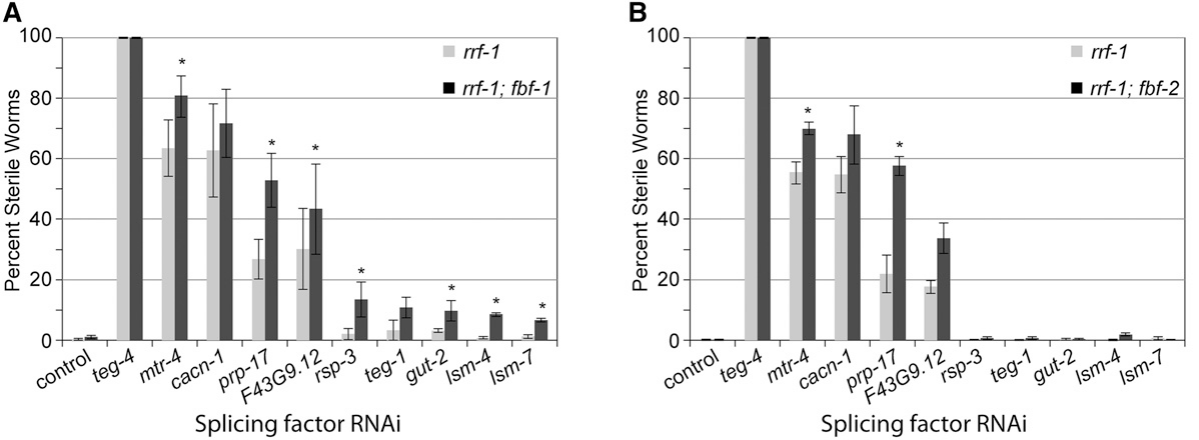
Splicing factor RNAi causes enhanced sterility of *fbf-1* and *fbf-2* mutants. The percentage of sterile hermaphrodites of the *rrf-1*, *rrf-1;fbf-1* (A) or *rrf-1;fbf-2* (B) genotype subjected to the indicated RNAi treatments. Sterile animals were identified by the absence of embryos in the uterus after 24 hr past the L4 larval stage. Error bars indicate SEM (from three or four experiments). Asterisks mark the treatments that caused significant increase in sterility of the double-mutant animals compared to the *rrf-1* mutant (Student’s paired *t*-test; *P* < 0.05).

To identify possible FBF-2 cofactors and additional genes involved in regulation of the proliferation/differentiation transition in the germline, we performed an RNAi enhancer screen of 16 candidate genes predicted to contribute to FBF-2−mediated regulation (www.geneorienteer.org; Zhong and Sternberg 2006) as well as a subset of 34 genes predicted to function in RNA regulation or metabolism and highly expressed during oogenesis (Reinke *et al.* 2004). The oogenesis-enriched RNA regulators tested in this study are a part of an ongoing large-scale genetic interaction screen. We assayed for enhanced sterility in the *fbf-1* mutant background compared with the control strain. Both strains carried a mutation in *rrf-1* to preferentially direct RNAi to germline tissues (Sijen *et al.* 2001; Kumsta and Hansen 2012). Knockdown of three splicing factors, *prp-17*, *lsm-4*, and *gut-2*, resulted in enhanced sterility when depleted in *rrf-1*; *fbf-1* mutant worms compared with the *rrf-1* strain (Figure 1A and data not shown). All three splicing factors were present in the list of predicted FBF-2 cofactors. *prp-17* and *gut-2* also belong to the complete oogenesis-enriched RNA regulator gene set that was analyzed only partially in this study, but likely also had potential to recover splicing factors. The rest of the tested clones (47) failed to show enhanced sterility resulting either in completely fertile worms in both genetic backgrounds or in equal percentages of sterile worms across tested genetic backgrounds. These results suggest that multiple components of the spliceosome genetically interact with the *fbf-1* mutant.

To test whether other components of the splicing machinery genetically interact with *fbf-1*, we used RNAi to deplete seven additional splicing factors distributed throughout the splicing reaction cycle. We chose the genes suggested in previous reports to function in splicing reaction and focused on those that have previously produced genetic interaction with *glp-1*, a regulator of germline proliferation (Mantina *et al.* 2009; Kerins *et al.* 2010). Knockdown of six of these genes resulted in enhanced synthetic sterility in the *rrf-1*; *fbf-1* mutant (which reached statistical significance in four cases), whereas knockdown of the seventh (*teg-4*) induced 100% sterility even in the *rrf-1* strain (Figure 1A). Collectively, seven distinct components of the spliceosome significantly interact with *fbf-1* and thus may contribute to FBF-2 function.

**Figure 2:**
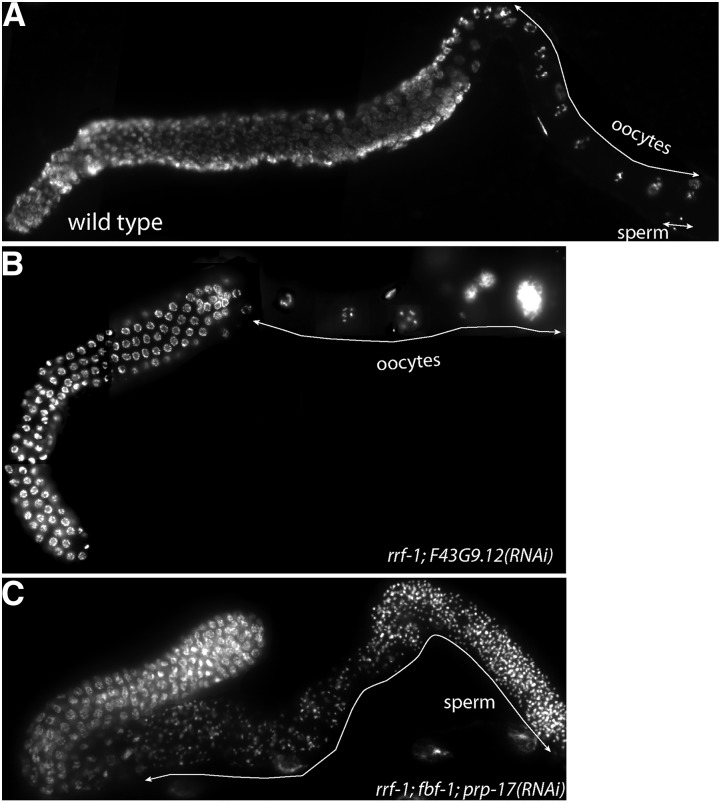
Germline masculinization after splicing factor knockdown. Full germlines were dissected and fixed, and chromatin was stained with DAPI. (A) Control treatment, wild-type germline. (B) *rrf-1*; *F43G9.12(RNAi)*, germline with degenerating endomitotic oocytes. (C) *rrf-1*; *fbf-1*; *prp-17(RNAi)*, masculinized germline. The control germline contains all stages of germ cell differentiation, including oocytes. By contrast, masculinized germline contains mainly spermatogenic cells.

We next tested whether the synthetic sterility in the RNAi assays phenocopied that of *fbf-1fbf-2* double mutants, which fail to transition from spermatogenesis to oogenesis (Crittenden *et al.* 2002). We determined gamete chromatin morphology in the three treatments (*mtr-4*, *F43G9.12*, and *prp-17(RNAi)*) that produced high levels of enhanced sterility in the *fbf-1* mutant background (Figure 1A). Similar to *fbf-1fbf-2* double mutants, the sterility of *rrf-1*; *fbf-1* worms after splicing factor depletion was associated with an increased prevalence of masculinized germlines (Figure 2C; Table 2), in contrast to the fertile germlines containing both oocytes and sperm (Figure 2A). The other sterile phenotype was associated with degenerated endomitotic oocytes (Figure 2B) and was more prevalent in the *rrf-1* background than in *rrf-1*; *fbf-1* background. This phenotype is not relevant to sex determination or *fbf* function. These observations suggest that splicing factors may contribute to *fbf-2* activity.

**Table 2:** Germline masculinization in sterile worms after splicing factor knockdown

RNAi	Strain		
	*rrf-1* %Mog (*n*)	*rrf-1*; *fbf-1* %Mog (*n*)	*rrf-1*; *fbf-2* %Mog (*n*)
Control day 1	0	0	0
day 3	0	0	0
*mtr-4* day 1	43% (23)	97% (33)	80% (45)
day 3	37% (27)	89% (35)	48% (31)
*prp-17* day 1	62% (42)	100% (33)	98% (64)
day 3	47% (15)	87% (46)	93% (29)
*F43G9.12* day 1	4% (23)	41% (34)	52% (46)
day 3	0% (26)	56% (34)	25% (56)

Germline masculinization was scored after staining of dissected gonads of sterile worms with DAPI if formation of sperm but not oocytes was detected. The animals were fixed and stained on day 1 post-L4 stage (3d) and on day 3 post-L4 stage (5d). In several treatments, percent masculinized germlines decreased on day 3 post-L4, suggesting that some but not all observed masculinization on day 1 post-L4 was attributable to a delay in the switch to oogenesis. Control RNAi treatments did not have sterile worms. (*n*), number of germlines scored.

To test whether splicing factors were selective for *fbf-2* or also contribute to *fbf-1* function, we tested whether the splicing factor RNAi is synthetically sterile with the *fbf-2* mutation. We found that knockdowns of two splicing factors, *mtr-4* and *prp-17*, produced significant synthetic sterility with *fbf-2* (Figure 1B). In contrast, knockdowns of five genes producing synthetic sterility with the *fbf-1* mutation (*rsp-3*, *teg-1*, *gut-2*, *lsm-4*, and *lsm-7*) failed to generate synthetic sterility with *fbf-2*, indicating either specific cooperation of these splicing factors with FBF-2 or a weaker overall FBF regulation in *fbf-1* mutant leading to a greater sensitivity to synthetic interactions. The synthetic sterility in *fbf-2* background was associated with an increased prevalence of masculinized germlines (Table 2). Together, these results suggest that the splicing machinery contributes to function of both FBF-1 and FBF-2, and depletion of splicing factors promotes sterility when either FBF-1 or FBF-2 are absent.

### Splicing factor RNAi affects FBF target regulation

**Figure 3:**
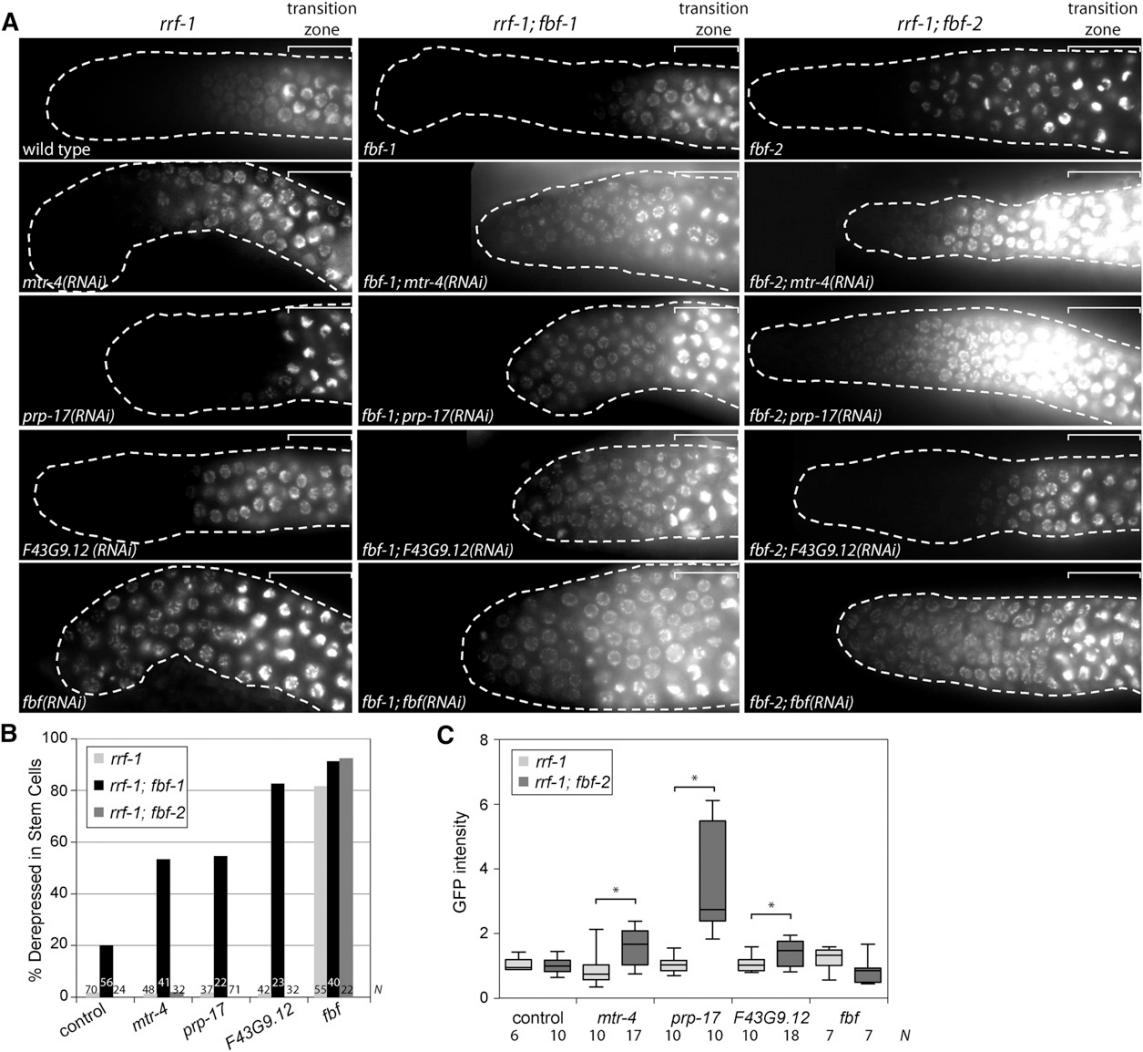
Derepression of FBF target genes upon splicing factor RNA interference (RNAi) in sensitized backgrounds. (A) Distal gonads of the indicated genotypes expressing a GFP::Histone H2B fusion under the control of the *fog-1* 3′UTR after RNAi of the indicated splicing factor genes. Gonads are outlined; white brackets indicate the position of the transition zone as recognized by the “crescent-shaped” chromatin. All images were taken with a standard exposure. (B) The percentage of *rrf-1* (light gray), *rrf-1;fbf-1* (black), or *rrf-1;fbf-2* (dark gray) gonads following indicated RNAi with GFP::H2B::*fog-1* 3′UTR expression extending to the distal end. *N*, number of germlines scored. (C) Background-corrected GFP intensity in transition zone nuclei (normalized to the average GFP intensity of control RNAi on *rrf-1* strain) plotted for *rrf-1* (light gray) and *rrf-1;fbf-2* (dark gray) gonads after indicated RNAi treatments. Box plot whiskers indicate the minimum and maximum intensity values. *N*, number of germlines scored. Asterisks mark the treatments that caused significant increase in the reporter intensity of the double-mutant animals compared to the *rrf-1* mutant (Student’s *t*-test; *P* < 0.01). Note that the difference between reporter fluorescence after *F43G9.12(RNAi)* in *rrf-1* and *rrf-1;fbf-2* backgrounds is significant, although the absolute value of the increase is small (1.4-fold) and no germlines have fluorescence values twofold higher than the control. GFP, green fluorescent protein.

Next, we directly tested whether splicing factor RNAi affects FBF function by observing the effect of splicing factor depletion on an FBF target gene *fog-1* (Thompson *et al.* 2005). Expression of a transgenic GFP::Histone H2B::*fog-1 3′UTR* reporter is silenced in the mitotic zones of wild-type, *fbf-1*, and *fbf-2* worms, but it becomes derepressed in the mitotic zones of *fbf-1fbf-2* double-mutant germlines (Merritt *et al.* 2008). Upon splicing factor knockdown, 40–80% of sterile *rrf-1*; *fbf-1* hermaphrodites derepressed *fog-1* 3′UTR reporter in the mitotic region (Figure 3, A and B). By contrast, control depletion of the splicing factors in the *rrf-1* background did not result in significant reporter derepression in the mitotic region. These results indicate that depletion of splicing factors compromises FBF-2 activity in *fbf-1* mutant background.

To determine whether the splicing factors affect FBF-1 activity, we repeated the same experiments in the *fbf-2* mutant background (Figure 3, A and B). Although no treatments derepressed the transgenic reporter in the distal-most stem cell region, *prp-17(RNAi)* and *mtr-4(RNAi)* resulted in a dramatic increase of *fog-1* 3′UTR reporter expression in the transition zone where the cells entered meiosis (Figure 3A). Transition zone nuclei expressing *fog-1* 3′UTR reporter in the *rrf-1;fbf-2* background had on average 1.6 to 3.5 fold more GFP signal compared to the transition zone nuclei of the control germlines (Figure 3C; *P* < 0.01, Student’s *t*-test). Thus, knockdown of splicing factors may limit FBF-1 activity in the *fbf-2* mutant background. These results are consistent with previous findings that splicing factors *mog-1* and *mog-6* repress expression of *fem-3* 3′UTR reporter in somatic cells (Gallegos *et al.* 1998).

### Splicing factor RNAi enhances feminization of *fog-1(ts)* mutant

Our results indicate that loss of splicing factors enhances the single *fbf* mutant phenotype and that, like the *fbfs*, splicing factors are required for stem cell maintenance. However, previous studies suggested that a decrease in splicing factor activity instead leads to the opposite phenotype: overproliferation and formation of synthetic germline tumors in combination with a weak gain of function allele of *glp-1* (Mantina *et al.* 2009; Kerins *et al.* 2010; Wang *et al.* 2012). Because of these opposing combinatorial effects, we hypothesize that the role of splicing factors in germline stem cell proliferation and differentiation extends beyond generating specific splice isoforms of the stem cell maintenance regulators. We suggest the splicing factors act more broadly to maintain efficient translational control of germline mRNAs.

To test whether splicing factors are broadly required for RNA regulation, we took advantage of the *fog-1(q253ts)* mutant, which leads to failure of sperm production at the restrictive temperature of 25° but permits spermatogenesis at 15° (Barton and Kimble 1990; Jin *et al.* 2001a). The level of FOG-1 expression is tightly controlled and correlates with sperm number produced by the hermaphrodite (Barton and Kimble 1990; Lamont and Kimble 2007); therefore, any defect in FOG-1 function would be manifested in decreased or absent sperm production. If the normal function of splicing factors is to act with the *fbfs* to promote oogenesis, splicing factor knockdown would still cause masculinization in the *fog-1(ts)* background at the permissive temperature, where FOG-1(ts) is functional. Alternatively, if splicing factor knockdown disrupts RNA regulation in general rather than selectively affecting *fbf* function, it would produce synthetic feminization of the *fog-1(ts)* mutant at the permissive temperature.

**Figure 4:**
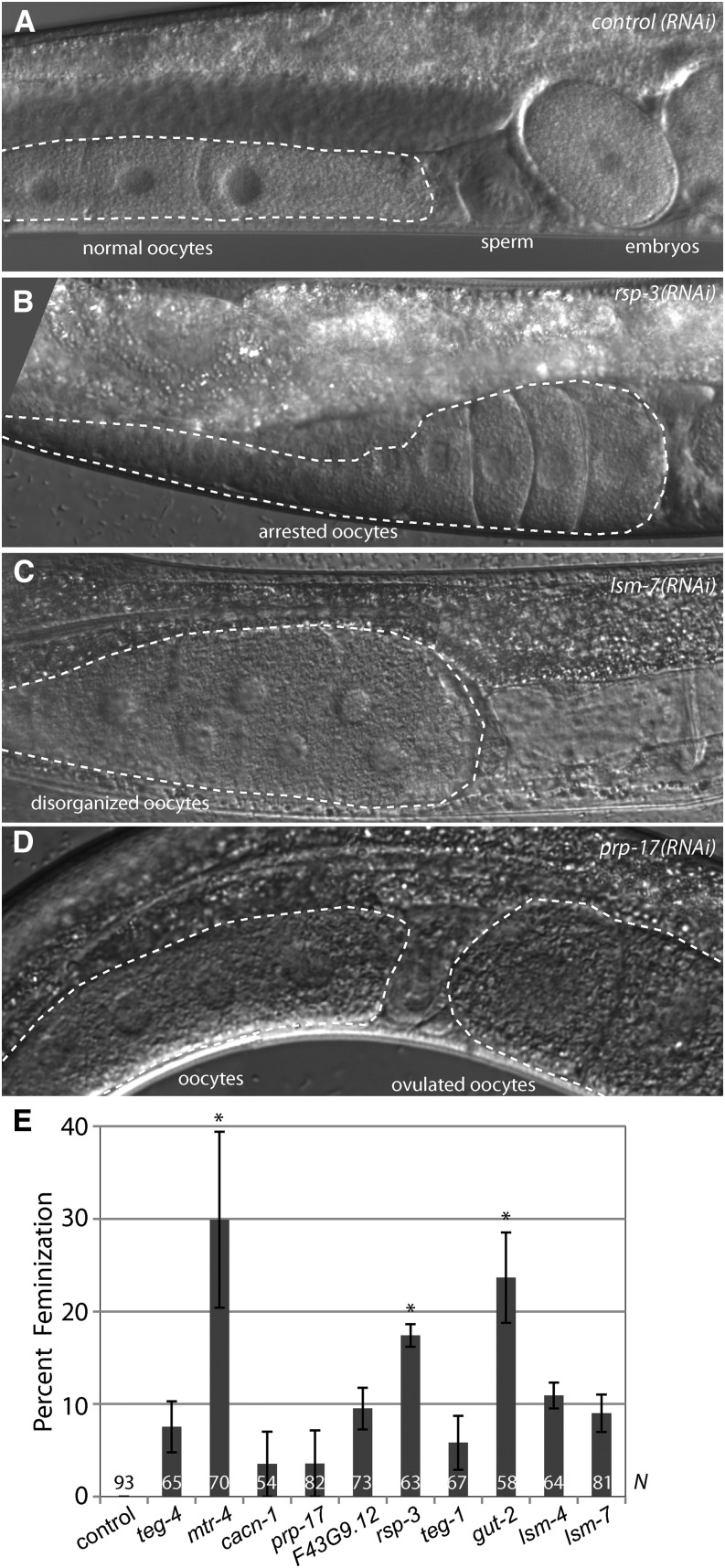
Defective spermatogenesis in *fog-1(ts)* mutants treated with splicing factor RNA interference (RNAi). (A) Normal germline, containing both oocytes and sperm. (B−D) A range of phenotypes caused by splicing factor RNAi in *fog-1(ts)* strain at permissive temperature includes arrested, disorganized, or ovulated oocytes. Each panel indicates the corresponding RNAi treatment. (E) The percentage of *fog-1(ts)* hermaphrodites showing spermatogenesis defects following indicated RNAi treatments. Error bars indicate SEM (from three or four experiments). Asterisks mark the treatments that caused significant increase in defective spermatogenesis compared to the control pL4440 RNAi (*P* < 0.05; corrected for multiple comparisons). Control and experimental groups were compared by one-way analysis of variance (*P* = 0.0002), followed by post-test comparison of treatments to control by the Dunnett multiple comparison test. *N*, number of hermaphrodites scored.

Knockdown of splicing factors at permissive temperature failed to masculinize *rrf-1fog-1(ts)* strain. By contrast, RNAi of all tested splicing factors in *rrf-1fog-1* background produced some level of synthetic feminization; this feminization reached statistical significance in three cases (Figure 4E). Feminized phenotypes included arrested oocytes characteristic of *fog-1* loss of function (sometimes disorganized) and ovulated unfertilized oocytes, indicating defects in spermatogenesis (Figure 4, B−D). In some cases, feminization was incomplete, and small amounts of sperm were produced before a switch to oogenesis detected by the presence of two to three embryos in the adult’s uterus followed by ovulated or arrested oocytes. None of these phenotypes was observed in *fog-1(ts)* worms exposed to the control RNAi, in nonmasculinized *rrf-1* mutant worms exposed to splicing factor RNAi, or in previous reports of splicing factor mutants. Because splicing factor knockdown may lead to either synthetic masculinization (*fbf* mutant background) or synthetic feminization (in *fog-1(ts)* background), the function of splicing factors in germline sex determination is not specific to the FBFs or oogenesis. Instead, we conclude that the functional splicing cascade facilitates RNA regulation carried out by multiple regulatory proteins in the germline.

### Splicing RNAi does not enhance embryonic lethality of *dhc-1(or195ts)*

One potential consequence of splicing factor knockdown is general deterioration of all cellular functions; in that case, it would be expected to worsen the phenotype of any loss-of-function mutation, especially those that affect cell viability. To test whether a partial loss of function mutation would be nonselectively enhanced by depletion of splicing factors, we tested our panel of splicing factor RNAi in a strain carrying the temperature-sensitive S3200L mutation in the motor subunit of dynein, *dhc-1(or195ts)* (Hamill *et al.* 2002). This mutation causes embryonic lethality at 25° because of failure of mitotic spindle alignment, chromosome congression defects, and mitotic spindle collapse within 1 min of temperature upshift; thus, the phenotype most likely does not involve changes in posttranslational regulation of gene expression (Schmidt *et al.* 2005). We expect that if splicing factor depletion causes nonspecific loss of viability and enhances reduction-of-function mutation phenotypes, the embryonic lethality of *dhc-1(ts)* would be enhanced at the permissive temperature. Conversely, if splicing factor depletion primarily affects RNA regulation, the embryonic lethality of *dhc-1(ts)* would be equal either to the lethality of untreated *dhc-1(ts)* or to the lethality of splicing factor-depleted wild-type control.

**Figure 5:**
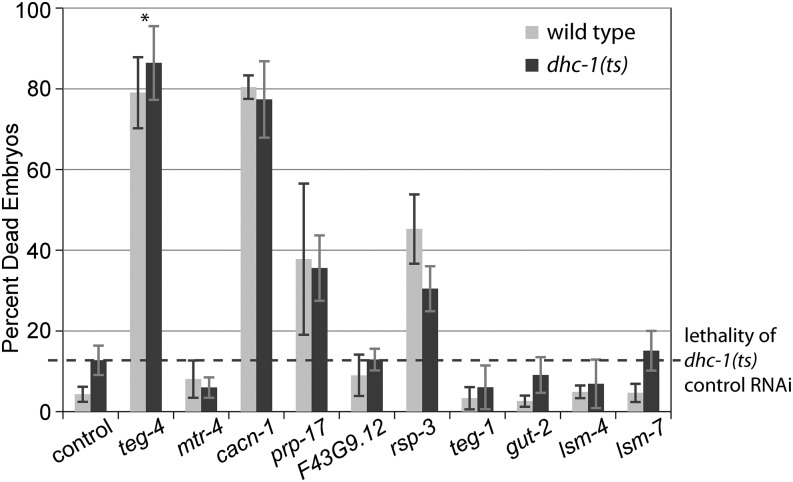
Splicing factor RNA interference (RNAi) does not produce synthetic lethality with *dhc-1(ts)* mutant. The percentage of dead embryos produced by N2 (wild type) or *dhc-1(ts)* hermaphrodites treated with indicated RNAi. Error bars indicate SEM (from two to four experiments). Asterisk marks *teg-4(RNAi)*, which caused a significant increase in embryonic lethality in *dhc-1(ts)* mutant compared to wild type control (Student’s paired *t*-test; *P* = 0.007).

RNAi knockdowns of *mtr-4*, *F43G9.12*, *lsm-4*, *lsm-7*, *gut-2*, and *teg-1* resulted in lethality similar to that observed in *dhc-1(ts)* treated with control RNAi. Knockdowns of *cacn-1*, *prp-17*, and *rsp-3* showed pronounced embryonic lethality, albeit equal between N2 and *dhc-1(ts)* strains treated with splicing factor RNAi (Figure 5). *teg-4(RNAi)* caused small but statistically significant enhancement of embryo lethality in the *dhc-1(ts)* mutant. Because the severity of the lethality caused by combined *teg-4(RNAi)* and *dhc-1(ts)* is close to the sum of the effects of the two perturbations individually, this effect appears additive rather than synthetic. We conclude that in the majority of cases splicing factor knockdowns do not exacerbate a developmental defect unrelated to RNA regulation.

## Discussion

Here, we demonstrate that reduction in the activity of the splicing pathway in *C. elegans* germline disrupts multiple processes that depend on posttranscriptional control of gene expression. This destabilization of RNA regulation is uncovered by genetic interaction assays that identify splicing factor knockdowns as genetic enhancers of partial loss-of-function mutations in RNA-binding proteins. We suggest that an important function of the splicing pathway is to facilitate RNA regulation in general, which includes regulation by PUF-family translational repressors FBFs. Regulation of germline stem cell balance between proliferation and differentiation as well as spermatogenesis to oogenesis transition is centered at the posttranscriptional level. Our hypothesis explains the observations that reduction of splicing factor function may exacerbate defects that lead to opposite phenotypic outcomes such as masculinization and feminization; or overproliferation and stem cell loss. In our study, the strains that are mutant for RNA-binding proteins don’t show sterility, sex determination, or reporter misexpression phenotypes unless splicing factors are knocked down. This suggests that the enhanced phenotypes resulting from a combination of RNA-binding protein mutation with splicing factor knockdown reflect a synthetic interaction rather than an additive effect.

Synthetic interactions observed in this and other studies likely do not result from missplicing of one specific transcript, because splicing factor knockdowns produce opposite synthetic phenotypes depending on the genetic background (tumor *vs.* loss of stem cells; masculinization *vs.* feminization). Indeed, so far, no specific missplicing events accounting for overproliferation or masculinization phenotypes of the majority of splicing factor mutants have been identified (Puoti and Kimble, 1999; Belfiore *et al.* 2004; Kasturi *et al.* 2010; Zanetti *et al.* 2011), although general defects in splicing have been suggested (Zanetti *et al.* 2011). Export of unspliced *tra-2* mRNA and aberrant cytoplasmic splicing resulting in accumulation of a dominant-negative protein is thought to cause masculinization after depletion of exon junction complex components *mag-1* and *Y14* (Shiimori *et al.* 2013). However, cytoplasmic leakage of unspliced *tra-2* mRNA was not a consequence of a general splicing defect, and was not observed upon depletion of other splicing factors.

Despite the essential contribution of splicing to gene expression, splicing factor knockdowns change gene expression patterns in germline rather than cause tissue degeneration. This is likely due to a partial loss-of-function produced by splicing factor RNAi treatments.

### Translational repression

The switch from spermatogenesis to oogenesis in the adult depends in part on translational repression of *fem-3* mRNA by FBF proteins (Zhang *et al.* 1997). Splicing factor genes *mog-1*, *mog-4*, and *mog-5* were isolated in the screen for mutations that disrupt the sperm to oocyte switch (Graham and Kimble 1993; Graham *et al.* 1993). A transgenic reporter expressed in the somatic tissues and regulated by *fem-3* 3′UTR was used previously to assess the role of *mogs* in the translational control of *fem-3* (Gallegos *et al.* 1998). In wild-type animals, the reporter was expressed only weakly, but in the *mog* mutant background, significant derepression was observed in somatic tissues. The conclusion that *mog* genes contribute to *fem-3* translational repression in the somatic tissues also was presumed true for the germline, although the mechanism of regulatory input by MOG proteins remained unclear (Gallegos *et al.* 1998).

We find that disruption of splicing factor genes by RNAi derepresses a germline-expressed *fog-1* transgenic reporter, which is normally silenced by FBF activity in stem cells. We observed two types of derepression: expression of the reporter throughout distal mitotic region and up-regulation of the reporter expression in meiotic cells (typically along with reporter expression in some but not all mitotic cells). Up-regulation of the *fog-1* reporter in meiotic cells is reminiscent of the regulation of another FBF target, FEM-3. Normally, FEM-3 is expressed in the primary spermatocytes, but several conditions disrupting *fem-3* regulation by the FBFs lead to an expansion of FEM-3 expression to pachytene, but not to the stem cell region (Zanetti *et al.* 2012). We observed *fog-1* reporter derepression in the backgrounds where one of two *fbf* genes was mutated, but rarely in the wild-type background worms subjected to splicing factor RNAi. We hypothesize that combined residual activity of FBF-1 and FBF-2 upon splicing factor depletion in the wild-type background is sufficient to maintain FBF-mediated target repression in germline stem cells. Why then did the previous study find somatic *fem-3* reporter derepression in splicing factor mutants despite the presence of both FBF-1 and FBF-2 (Gallegos *et al.* 1998)? Both FBFs are predominantly expressed in the germline, and the baseline somatic activity of these proteins is much lower than the germline activity. This marginal activity of FBFs that represses *fem-3* 3′UTR reporter in somatic tissues is further reduced by mutation in splicing factors causing *fem-3* reporter derepression. By contrast, in germline, the level of FBF protein and activity are greater, so that one of the genes has to be mutated for the splicing factor RNAi to have an effect. Combined, our and previous results suggest that deficient splicing activity leads to disruption of translational control by FBFs.

### Splicing factors and sex determination

One of the synthetic phenotypes observed upon splicing factor RNAi in the *fbf* mutant background is masculinization of the germline. Germline masculinization was reported for single mutants of several splicing factors, including *prp-17* (Kerins *et al.* 2010). In addition, we observed synthetic masculinization after *mtr-4(RNAi)* and *F43G9.12(RNAi)*, that were not reported to produce masculinization when depleted singly (Kerins *et al.* 2010). If splicing machinery were specifically required to work with FBFs (directly or indirectly), splicing factor RNAi would result in masculinization independent of genetic background. Instead, we observed that splicing factor RNAi of *fog-1(ts)* animals at the permissive temperature was associated with weak but significant synthetic feminization of germline indicative of *fog-1* loss of function. We hypothesize that the temperature-sensitive mutation in the RNA-binding domain of FOG-1 renders it sensitive to the ribonucleoprotein (RNP) assembly defects resulting from inefficient splicing activity. Previous studies of splicing factors in sex determination found that feminizing null mutations in *fog-1*, *fog-3*, and *fem-3* are epistatic to masculinization of germline observed in splicing factor mutants (Graham and Kimble 1993; Kerins *et al.* 2010; Wang *et al.* 2012). Genetically, it suggests that splicing factors function upstream of the *fog/fem* genes. However, we find that knockdowns of splicing factors instead enhance weak *fog-1* mutation, suggesting that in addition to regulating FOG-1 production, splicing machinery is important for FOG-1 function.

### How do splicing factors contribute to gene regulation?

We propose that the splicing process contributes to efficient posttranslational control of mature spliced mRNA. Disruption of the splicing cascade may lead to defects in the assembly of messenger RNPs, which then fail to undergo normal cytoplasmic regulation. Therefore, the effects of mild splicing disruption will be most pronounced in systems heavily reliant on the posttranscriptional control of gene expression, such as *C. elegans* germline, and readily manifest in the sensitized mutant backgrounds. Some splicing factors remain associated with the spliced transcript, such as the exon junction complex, or EJC (Kataoka *et al.* 2000; Le Hir *et al.* 2000, reviewed in Le Hir and Séraphin 2008). Although the core of the EJC persists during RNP maturation, peripherally associated components change as the messenger RNP is exported from the nucleus and regulated in the cytoplasm. Splicing-dependent deposition of the EJC plays a profound role in mRNA metabolism, regulating nuclear export, nonsense-mediated decay, efficiency of translation, and RNA localization (Hachet and Ephrussi 2004; Ghosh *et al.* 2012, 2014; Popp and Maquat 2014). One possibility is that deposition of EJC or similar complexes is disrupted by the treatments reducing overall splicing efficiency.

### Splicing factor knockdown specifically enhances mutations affecting RNA regulation

Our results suggest that down-regulation of splicing pathway enhances the phenotypes caused by defects in RNA regulation but not embryonic lethality resulting from disruption of cytoplasmic dynein. Similarly, a whole-genome synthetic interaction screen for genes contributing to function of *mel-28* failed to retrieve splicing factors as genetic interactors (Fernandez *et al.* 2014). MEL-28 is a conserved component of nuclear pores needed for reestablishment of nuclear envelope after cell division and is not expected to contribute to RNA regulation. In the same vein, mutation in splicing factor *teg-4* does not enhance weak *lin-12* mutations interfering with Notch signaling in the anchor cell/vulval precursor cell fate decision, despite showing genetic interactions with pathways regulating the balance between germ cell proliferation and differentiation (Mantina *et al.* 2009). By contrast, splicing factors were isolated as enhancing the phenotype of *lin-35* Retinoblastoma homolog (Ceron *et al.* 2007), whose regulatory targets are under extensive posttranscriptional control (Grishok and Sharp 2005; Grishok *et al.* 2008). Additionally, splicing factors were isolated in synthetic screens for the enhancers of germline overproliferation phenotype in the sensitized backgrounds of weak *glp-1(gf)* (Mantina *et al.* 2009; Kerins *et al.* 2010; Wang *et al.* 2012). Together, these data suggest that the processes involving RNA regulation are likely to produce genetic interaction with splicing factors.

The broad contribution of splicing to posttranscriptional control needs to be taken into account when interpreting results of large-throughput genetic enhancer screens. We recommend to take genetic screen results identifying splicing factors as enhancers of a particular mutant phenotype as an indication that posttranscriptional gene regulation plays a major role in the process under investigation. However, in absence of other supporting evidence, genetic interaction most likely reflects a broad role for the splicing factors in maintaining efficient RNA regulation rather than specific contribution to the function of the gene mutated to sensitize a strain to genetic interaction.
